# Multi-polygenic score prediction of mathematics, reading, and language abilities independent of general cognitive ability

**DOI:** 10.1038/s41380-024-02671-w

**Published:** 2024-07-31

**Authors:** Francesca Procopio, Wangjingyi Liao, Kaili Rimfeld, Margherita Malanchini, Sophie von Stumm, Andrea G. Allegrini, Robert Plomin

**Affiliations:** 1 Social, Genetic and Developmental Psychiatry Centre, Institute of Psychiatry, Psychology and Neuroscience, King’s College London, London, UK; 2 School of Biological and Behavioural Sciences, Queen Mary University of London, London, UK; 3 Department of Psychology, Royal Holloway, University of London, Egham, Surrey, UK; 4 Department of Education, University of York, York, UK; 5 Department of Clinical, Educational and Health Psychology, Division of Psychology and Language Sciences, University College London, London, UK

**Keywords:** Genetics, Psychology

## Abstract

Specific cognitive abilities (SCA) correlate genetically about 0.50, which underpins general cognitive ability (g), but it also means that there is considerable genetic specificity. If g is not controlled, then genomic prediction of specific cognitive abilities is not truly *specific* because they are all perfused with g. Here, we investigated the heritability of mathematics, reading, and language ability independent of g (SCA.g) using twins and DNA, and the extent to which multiple genome-wide polygenic scores (multi-PGS) can jointly predict these SCA.g as compared to SCA uncorrected for g. We created SCA and SCA.g composites from a battery of 14 cognitive tests administered at age 12 to 5,000 twin pairs in the Twins Early Development Study (TEDS). Univariate twin analyses yielded an average heritability estimate of 40% for SCA.g, compared to 53% for uncorrected SCA. Using genome-wide SNP genotypes, average SNP-based heritabilities were 26% for SCA.g and 35% for SCA. We then created multi-PGS from at least 50 PGS to predict each SCA and SCA.g using elastic net penalised regression models. Multi-PGS predicted 4.4% of the variance of SCA.g on average, compared to 11.1% for SCA uncorrected for g. The twin, SNP and PGS heritability estimates for SCA.g provide further evidence that the heritabilities of SCA are not merely a reflection of g. Although the relative reduction in heritability from SCA to SCA.g was greater for PGS heritability than for twin or SNP heritability, this decrease is likely due to the paucity of PGS for SCA. We hope that these results encourage researchers to conduct genome-wide association studies of SCA, and especially SCA.g, that can be used to predict PGS profiles of SCA strengths and weaknesses independent of g.

## Introduction

For over a century, psychologists have developed hundreds of cognitive performance measures and several taxonomies of cognitive abilities. One of psychology’s most replicated and accepted findings is that all cognitive abilities substantially correlate with one another [1]. The shared variance between cognitive abilities is known as general cognitive ability (g). A widely accepted taxonomy of cognitive abilities is the Cattell-Horn-Carroll (CHC) hierarchical model of intelligence [2]. The CHC model positions g at the top of the three-stratum model, representing what is in common among 16 factors at the middle level of the model, such as quantitative knowledge, reading and writing and processing speed. These broad factors encompass clusters of scores of correlated cognitive measures that comprise the lowest level. By tradition, we refer to the middle level of the CHC model as specific cognitive abilities (SCA), even though these factors are not independent of g.

Family, twin, and adoption studies have consistently found that individual differences in both g and SCA are substantially heritable [3]. A recent meta-analytic review of 747,567 monozygotic-dizygotic twin comparisons reported that SCA are, on average, 56% heritable, which is similar to the 50% estimate typically found for g [4]. Moreover, it is well established that the genetic influences that contribute to individual differences in SCA substantially covary among SCA with genetic correlations consistently about 0.50 among diverse SCA [1]. Nonetheless, no genetic correlations near 1.0 have been reported, indicating that SCA have a unique genetic component and do not solely reflect the heritability of g.

The few studies that have attempted to investigate the unique genetic component of SCA suggest a surprising finding: the heritabilities of SCA phenotypically corrected for g (SCA.g) via regression are substantially heritable, 53% on average, very similar to the average heritability estimate of 56% for the same measures of SCA uncorrected for g [4].

The high heritability estimates of SCA and SCA.g make them good targets for genome-wide association (GWA) analysis, which identifies associations between DNA variants (single nucleotide polymorphisms, SNPs) and a target complex trait. The effect sizes of single SNP associations with complex traits are extremely small, the largest accounting for less than 0.05% of the variance, but these effects can be aggregated into polygenic scores (PGS) that can be used as genetic predictors of SCA [5]. The development of powerful polygenic scores of SCA would enable the creation of genetic profiles of strengths and weaknesses of cognitive abilities from birth. For instance, a polygenic score for reading could be used to detect risk for reading problems and enable interventions to forestall problems rather than waiting for them to emerge in school. However, due to the high genetic correlation between SCA and g, much of the prediction of reading would be due to g rather than reading per se, the unique aspect of the SCA. In order to develop genetic profiles of SCA independent of g, GWA studies of SCA.g are required.

To date, only one GWA study of SCA.g has been published. Donati et al [6] conducted a GWA study of English, maths and science independent of g, and reported significant SNP heritabilities for maths (24%) and science (15%). However, the sample sizes used were too small to detect significant genome-wide significant SNPs (maximum N = 3260). Moreover, the sample sizes of early GWA studies of SCA were too small—fewer than 10,000 individuals and often fewer than 1000 – to identify replicable associations for the very small effect sizes that we now know are responsible for the heritability of complex traits [7–16]. As a result, few genome-wide significant associations were found. Sample sizes in the hundreds of thousands are required to identify SNP associations of the expected effect size. Indeed, a recent GWA study of five reading and language traits with sample sizes of up to 34,000, but with a wide age range from 6 to 26 years, found only one genome-wide significant association with one trait [17].

These GWA studies of SCA did not calculate PGS from their GWA summary statistics to ascertain the power of their PGS to predict their target traits in independent samples. However, because the predictive power of PGS is correlated with the number of genome-wide significant associations, their PGS are not likely to predict much variance in the target traits. This trend can be seen in other GWA studies of SCA that reported the predictive power of PGS derived from their GWA summary statistics [18–21]. In contrast, a GWA study of self-reported mathematics performance in secondary school with sample sizes of about 500,000 from 23andMe yielded PGS that predicted an average of 6% of the variance in an independent sample [20]. Similarly, a PGS derived from the latest GWA study of the extremely broad trait of educational attainment with a sample size of three million predicted 12.4% of the variance for verbal grade point average (GPA), 10.0% for science GPA and 8.4% for mathematics GPA [22].

In summary, in order to develop genetic profiles of strengths and weakness of SCA.g, large GWA studies of SCA.g are needed. Until then, we can use extant PGS jointly in a multi-PGS strategy to increase the proportion of variance predicted by SCA and SCA.g. Analogous to the empirical approach used to create PGS by aggregating SNPs as long as they add to the prediction of the GWA target trait, a multi-PGS approach aggregates diverse PGS as long as they add to the prediction of the target trait [23]. We can widen this multi-PGS net beyond cognitive-related PGS to include PGS for personality and mental health traits that tap into noncognitive aspects of these abilities [24]. Finally, we can push this multi-PGS approach to its agnostic limit by including PGS for traits whose genetic relevance to cognitive traits is at best speculative, such as sub-cortical brain volumes and physical health.

In this study, we used an inclusive multi-PGS approach to maximise the prediction of mathematics, reading and language SCA and SCA.g assessed at age 12 in the Twins Early Development Study (TEDS) [25, 26]. We frame these genomic analyses in the context of heritability estimates of these same measures of SCA and SCA.g from twin analyses and SNP-based methods. This study was preregistered with the Open Science Framework (OSF; https://osf.io/jxbz8/).

## Methods

### Sample

Our sample was obtained from the Twins Early Development Study (TEDS) [25, 26]. The TEDS is a longitudinal twin study, which recruited over 16,000 twin pairs born in England or Wales between 1994 and 1996. Currently, over 8000 families still participate in the study, and they remain representative of the English and Welsh population in terms of ethnicity and socio-economic status for their birth cohort. The TEDS has collected a wealth of data over multiple time points, including data on the participants’ environment, physical wellbeing, personality, cognitive ability, and educational achievements. In addition, genomic data is available for over 10,000 twins.

In all our analyses, we excluded participants if they had a serious medical condition that could impact their ability to take part in TEDS assessments, severe problems surrounding birth which may have affected their development, or if important background information was missing. The sample sizes of each SCA and SCA.g by zygosity for the twin and genomic analyses is shown in Supplementary Table S1. We combined same and opposite-sex dizygotic twins in all our analyses because previous analyses indicated that sex differences accounted for little variance [27].

### Cognitive measures

At age 12, the twins completed a broad battery of 14 internet and telephone-based cognitive assessments, described below. Details of the assessment procedure and measures can be found in the TEDS data dictionary (https://www.teds.ac.uk/datadictionary/home.htm).

We constructed composite measures from the SCA described by Davis et al. [27]. Composite measures were created from standardised scores for reading ability (mean of four measures), mathematical ability (mean of three measures), language ability (mean of three measures) and g (mean of four measures). The composite measures were corrected for age and sex using standardised residuals.

#### Reading ability

To assess reading ability, the twins completed two online reading comprehension tests and two reading fluency tests, one online and the other via telephone. The two reading comprehension tests included an adaptation of the Peabody Individual Achievement Test [28] and the GOAL Formative Assessment in Literacy for Key Stage 3 [29]. The two reading fluency tests included an online adaptation of Woodcock- Johnson III Reading Fluency Test [30] and the Test of Word Reading Efficiency (TOWRE) [31], which was administered via telephone.

#### Mathematical ability

To assess mathematical ability, the twins completed three tests from the National Foundation for Education Research (NFER) booklets 6 to 14 [32]: Understanding Numbers, Non-numerical Processes and Computation, and Knowledge.

#### Language ability

Language ability was assessed using three online language tests of syntax, semantics and pragmatics. The test of syntax was the Listening Grammar subtest of the Test of Adolescent and Adult Language (TOAL-3) [33]. The semantics test was Level 2 of the Figurative Language subtest of the Test of Language Competence [34]. Pragmatics was assessed by the Level 2 of the Making Inferences subtest, Language Competence [34].

#### General cognitive ability (g)

Rather than extracting a latent variable from the 10 cognitive tests described above, g was assessed independently using four online tests, two verbal reasoning tests and two non-verbal reasoning tests [27]. The two verbal reasoning tests consisted of the WISC-III-PI Multiple Choice Information (General Knowledge) and the Vocabulary Multiple Choice [35]. The non-verbal tests included the WISC-III-UK Picture Completion [35] and the Raven’s Standard and Advanced Progressive Matrices [36, 37]. By deriving g from these four independent tests, we were able to create a balanced g factor and avoided overcorrecting the SCA for education-related tests of reading, mathematics and language. However, as a comparison, we also created a g factor from all 14 tests.

#### g-corrected SCA (SCA.g)

To construct the SCA.g measures, we regressed the g factor from reading, mathematical and language ability and used the standardised residuals as indices of SCA independent of g. A correlation matrix between the SCA, SCA.g, and *g* can be found in Supplementary Fig. S1. Each of the newly constructed SCA.g correlated strongly with their respective SCA (0.76 – 0.81), but weakly with the other two uncorrected SCA (0.22–0.29).

We also regressed the g factor derived from all 14 cognitive measures. Supplementary Table S2 shows a correlation matrix between uncorrected SCA, the 4-test g factor, the 14-test g factor, SCA corrected for the 4-test g factor, and SCA corrected for the 14-test g factor. The two g factors correlate highly (0.82). We focus our presentation of results on the 4-test g factor to avoid overcorrection, but results for twin and elastic net regression analyses for reading, mathematics and language ability corrected for the 14-test g factor are included in Supplementary Table S2.

### Twin analyses

#### Twin analyses

The twin method was used to explore the genetic and environmental aetiology of the SCA and SCA.g. Genetic and environmental components of a complex trait can be estimated by taking advantage of the quasi-experimental design provided by twins. Assuming additive genetic effects, monozygotic (MZ) twins share 100% of their inherited DNA variants, while dizygotic (DZ) twins share on average 50% of their DNA variants that vary between people. Both MZ and DZ twins are assumed to share 100% of their shared environment and 0% of their non-shared environment. By comparing MZ and DZ correlations, this standard twin model enables estimates of additive genetic (A), shared environmental (C) and non-shared environmental (E) effects. These ACE components of variance can be estimated by Falconer’s formula, which assumes an additive model. A is calculated as 2(rMZ-rDZ), C is estimated as residual MZ resemblance not explained by A (i.e., rMZ – A), and E is the remaining variance (1 – rMZ). These estimates can be more accurately and elegantly estimated by structural equation modelling [38]. We used maximum-likelihood model-fitting in OpenMx for R to test the fit of the univariate model and to estimate A, C and E parameters and their confidence intervals [39].

### Genomic analyses

#### SNP-based heritability

In addition to using a twin design to estimate heritability of each SCA and SCA.g, we also used genomic data to calculate SNP-based heritabilities. Details of the TEDS genotyping procedures can be found in the supplementary material of a previous TEDS paper by Selzam et al. [40] as well as in the TEDS data dictionary (https://www.teds.ac.uk/datadictionary/studies/dna.htm).

SNP heritability estimates the variance explained by all the SNPs included in genome-wide genotyping [41]. It represents the upper limit for variance explained by a PGS. In order to maximise the sample size of our genomic sample, we estimated SNP heritability using the method proposed by Zaitlen et al. [42] as it allows for the inclusion of family data, fraternal twins in our case.

We used the software, Genome-Wide Complex Trait Analysis (GCTA) to conduct our analyses [43]. For each SCA and SCA.g, two matrices were created. The first was a genomic relationship matrix (GRM_g_), which estimated the identity by state (IBS) of all pairs of individuals in the dataset. The second matrix was the kinship relationship matrix (GRM_k_). It was derived from the initial GRM_g_ in which the off-diagonals below 0.05 were set to 0. Restriction maximum likelihood (REML) implemented using GCTA was then applied to estimate the SNP-based and pedigree-based heritability from the two GRMs. We used the first 10 principal components and sex as covariates.

#### Polygenic scores (PGS)

PGS were constructed for each of the genotyped participants in the TEDS sample. The construction and quality control procedures are documented in previous papers published by the TEDS team [23, 40] and in the TEDS data dictionary: https://www.teds.ac.uk/datadictionary/studies/measures/polygenic_scores.htm. The genome-wide polygenic scores were previously derived using LDpred, and, as of 2022, are now derived using LDpred2-auto [44, 45]. All PGS used all SNPs (i.e., p-value threshold of 1 for SNP selection) and were corrected for the first 10 principal components, batch, and type of SNP chip.

We applied an empirical approach in the selection of PGS for our analyses. We began with 327 PGS available in the TEDS data dictionary as of 1^st^ October 2023 that met our inclusion criteria (https://www.teds.ac.uk/datadictionary/studies/measures/polygenic_scores.htm). We excluded PGS derived from GWA discovery samples with fewer than 10,000 individuals and PGS from GWA that included TEDS participants. In addition, due to 23andMe’s proprietary restrictions, we were not able to use GWA summary statistics that included 23andMe participants. These exclusions left us with 230 PGS.

A sample size of 10,000 individuals was used as a further criterion for selecting studies as this provides 80% power (alpha =0.05) to detect a correlation of 0.03, which is the largest effect size expected (i.e., r^2^ < .001). We correlated each of the 230 PGS with the three uncorrected composite SCA scores (mathematics, reading and language). For each composite, we excluded PGS that correlated less than 0.03 with them (Supplementary Table S3). We then used a multi-PGS approach to predict each of the three scores, as described in the next section. Supplementary Table S3 lists the PGS included in each approach, and Supplementary Table S4 provides general information on the PGS we included in this study.

#### Multi-PGS models

In order to maximise the prediction of SCA and SCA.g, we constructed multi-PGS scores to investigate the joint ability of PGS to predict the SCA and SCA.g measures [23]. Due to the large number of correlated predictors, we used elastic net penalised regression models with out-of-sample comparisons to reduce the number of PGS predictors and to provide unbiased estimates of predictive power [46]. Elastic net combines two types of regularisation: L1 regularisation (Lasso) and L2 regularisation (Ridge). Lasso encourages sparsity by reducing the number of predictors, and Ridge discourages extreme coefficient values by reducing the value of large regression coefficients.

We ran the elastic net regression models using the R package glmnet and caret [46, 47]. In all the models, the samples were split into an independent training set (80%) and a hold-out set (20%). In the training set, we performed 10-fold cross-validation repeated 100 times to select the model that minimised the Root Mean Square Error. We estimated variance explained (R^2^) in the hold-out set.

For comparison, we also conducted parallel standard multiple regression analyses for each of the SCA and SCA.g.

## Results

### Descriptive statistics

Means and standard deviations of the SCA and SCA.g are presented in Supplementary Table S5. Sex differences were calculated for both samples, and zygosity group differences (MZ and DZ) were also compared in the twin sample. To test for group differences, we conducted analysis of variance (ANOVA) of zygosity and sex and, although we found some significant differences (Supplementary Table S6), the differences accounted for less than 1% of the variance. All the measures in the subsequent analyses were corrected for age and sex, as twins correlate perfectly for age and same-sex twins also correlate perfectly for sex, which will inflate twin correlations for same-sex pairs [48].

### Twin analyses

**Fig. 1:**
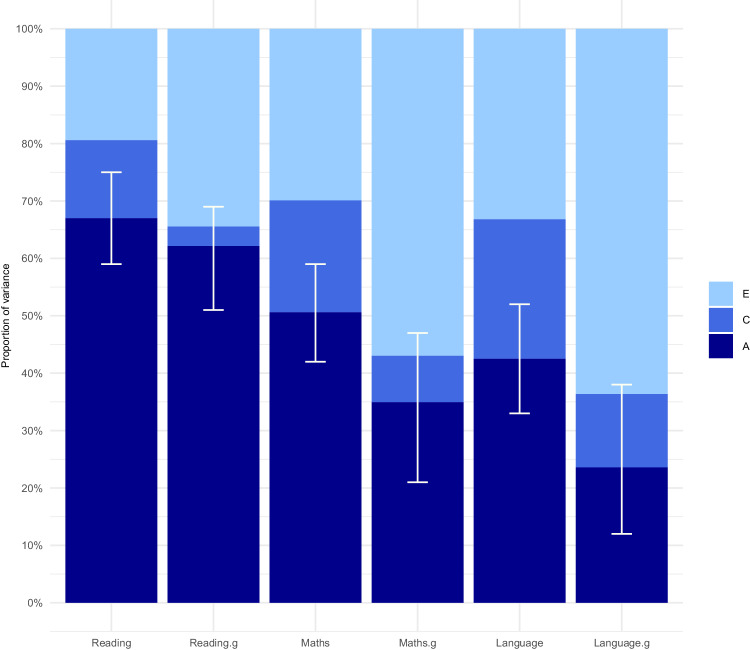
ACE twin results. The additive genetic, shared environmental and non-shared environmental components of reading, mathematics and language ability uncorrected for g (SCA) and corrected for g (SCA.g) at age 12. A= additive genetic influences; C= shared environmental influences; E= non-shared environmental influences. 95% confidence intervals for the additive genetic component are shown in the figure.

Figure 1 presents the ACE results from our twin model-fitting analyses. Point estimates and confidence intervals are in Supplementary Table S7. All the measures were found to be substantially heritable. The average heritability estimate is 53% for the uncorrected SCA and 40% for SCA.g, although none of these SCA and SCA.g heritabilities differed significantly. The order of heritability is the same for the SCA and SCA.g: heritability is highest for reading and lowest for language, with mathematics in the middle. C estimates were consistently lower for SCA.g than for SCA. The ACE estimates calculated by Falconer’s formula applied to twin intraclass correlations are nearly identical to the model-fitting ACE estimates in Fig. 1 (see Supplementary Table S8).

### SNP heritability

**Fig. 2:**
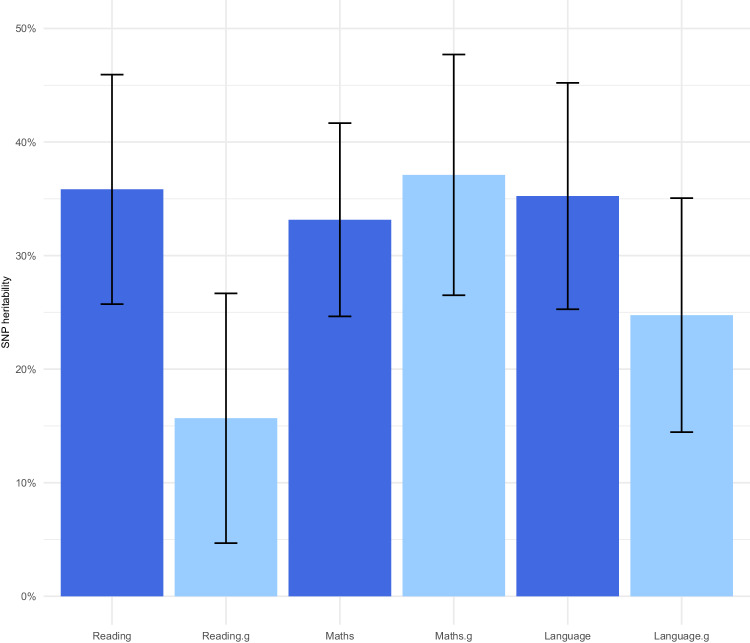
SNP heritabilities. SNP heritability of reading, mathematics and language ability corrected and uncorrected for g with standard errors as error bars.

The SNP heritabilities for each SCA and SCA.g are presented in Fig. 2. All SCA and SCA.g are significantly heritable. We observed a similar trend as in our twin analyses: the average SNP heritability of the SCA is higher (35%) than the average SNP heritability of the g-corrected SCA.g (26%). In other words, for both twin and SNP heritability, SCA.g are about 75% as heritable as SCA. The overlapping standard errors for these SNP heritability estimates indicate that differences between each pair of SCA and SCA.g are not significant, including the slightly higher SNP heritability for g-corrected mathematics (37.1%) as compared to uncorrected mathematics (33.2%).

### PGS predictions

Based on the criterion that a PGS correlate at least 0.03 with an SCA, 57 PGS were selected for the multi-PGS analysis of reading ability, 52 for mathematical ability and 50 for language ability. These correlations are reported in Supplementary Table S3. The highest correlations involved the 2022 PGS for educational attainment (EA4) [22], which correlated 0.31, 0.28, and 0.28, respectively, with reading, mathematical and language ability. All PGS generally correlated similarly with the three SCA.

**Fig. 3:**
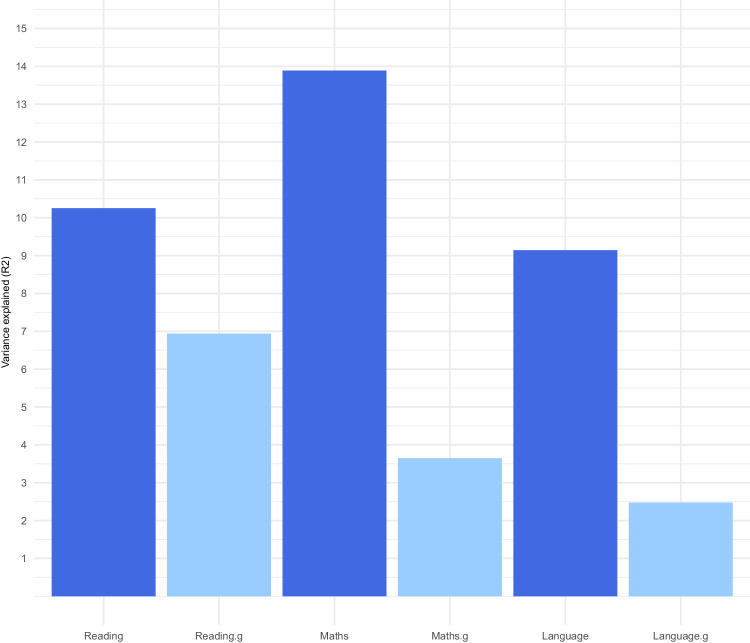
Multi-PGS results. Variance explained (R^2^) from the multi-PGS elastic net penalised regression models of reading, mathematics and language ability uncorrected (SCA) and corrected (SCA.g) for g.

The key finding is that multi-PGS significantly predicted all three SCA.g, although multi-PGS predicted significantly more variance for SCA uncorrected for g (Supplementary Table S9). Figure 3 shows the variance predicted from multi-PGS elastic net penalised regression models. The average variance explained was 11.1% for SCA and 4.4% for SCA.g. We reran the analyses with only one genotyped individual per DZ twin pair and found similar estimates (average SCA = 9.6%; average SCA.g = 4.3%) (Supplementary Table S10). In addition, simple multiple regression analyses yielded a similar pattern of results (average SCA = 10.1%; average SCA.g = 4.0%) (Supplementary Table S11).

As expected from the simple correlations (Supplementary Table S4), the EA4 PGS provided the strongest independent prediction of all three SCAs. EA4 PGS was also the strongest independent predictor of SCA.g for language ability, but a PGS for intelligence [49] was the strongest independent predictor of SCA.g for reading.g and a PGS for cognitive performance [20] was the strongest independent predictor for mathematics.g.

The multi-PGS approach predicted only slightly more variance than the single most predictive PGS (Supplementary Table S12).

**Fig. 4:**
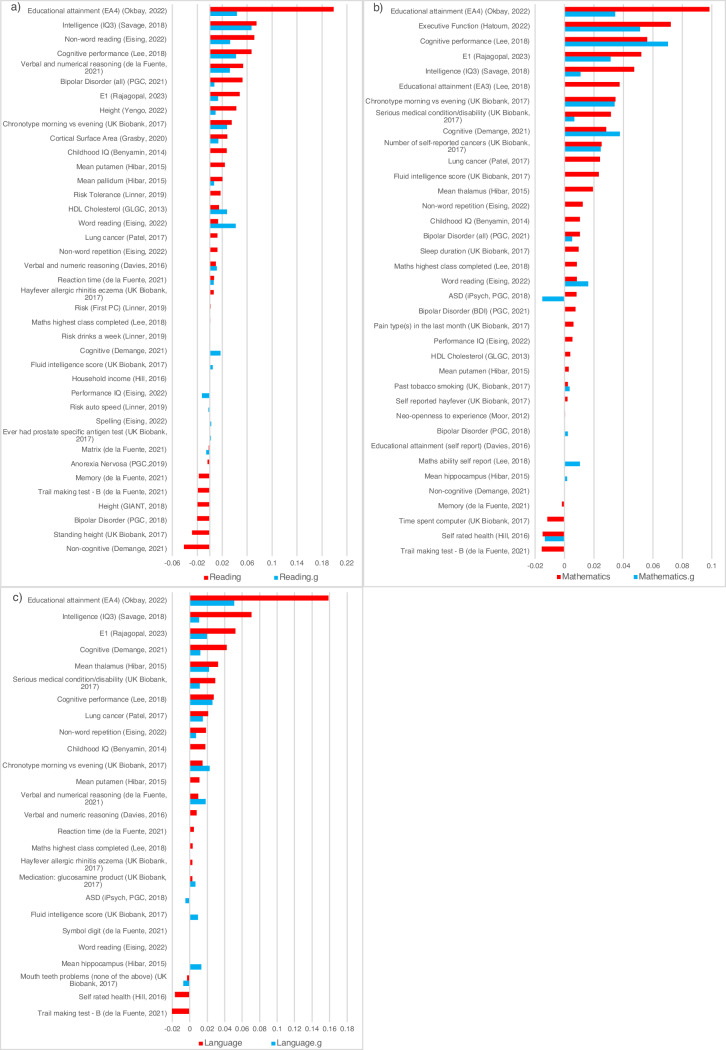
PGS contributions to multi-PGS prediction. Standardised coefficients from elastic net penalised regression model predicting: (**a**) uncorrected reading ability (Reading) and g-corrected reading ability (Reading.g). **b** Uncorrected mathematical ability (Maths) and g-corrected mathematical ability (Maths.g). **c** Uncorrected language ability (Language) and g-corrected language ability (Language.g). Only PGS that contributed to the prediction of the outcome are displayed here. Supplementary Table S3 shows results for all the predictors included in the model.

The standardised coefficients for each of the PGS from the multi-PGS elastic net penalized regression are shown in Fig. 4 for SCA and for SCA.g. The number of PGS retained in these regression analyses was 32, 33 and 22 for the three SCA, respectively, and 23, 17 and 19 for the three SCA.g. Squaring these coefficients, the largest independent contributions only explain 4% of the variance. Nonetheless, although the independent predictions from the other PGS are small, they add to the predictive power of the multi-PGS.

## Discussion

Our twin analyses revealed that the twin heritabilities of reading, mathematics and language ability independent of g (SCA.g) are significant and substantial (average 40%), although lower than SCA uncorrected for g (average 53%). In our previous meta-analytic review of SCA and SCA.g, the average heritability estimates were more similar: 53% for SCA.g and 56% for SCA [4]. We hypothesise that our current estimates of the heritability of SCA.g are more accurate, because they are based on an extensive battery of four tests of g, four reading tests, three mathematics tests and three language tests. The meta-analytic average heritability of SCA.g was instead based on the previous literature on SCA.g which consists of only three studies. One study examined academic performance, rather than cognitive ability, at age 16 [50], and the other two each investigated a single domain of ability—mathematics [51] and spatial ability [52]. Regardless, the average twin heritability of the SCA.g investigated here is substantial and the differences in heritability between the respective g-corrected and uncorrected SCA is non-significant. In addition, we investigated the twin heritability of reading, mathematics and language ability corrected for a g factor derived from all 14 cognitive assessments and found that the confidence intervals between all the corresponding SCA.g overlap when corrected for the 4-test g or the 14-test g (Supplementary Table S2).

We also observed significant and substantial SNP heritabilities for the three SCA.g. As with the twin heritability estimates, we found that SNP heritability estimates for SCA.g were about 75% of the SNP heritabilities for SCA (26% vs 35%).

The drop in heritability from SCA to SCA.g is more pronounced in our multi-PGS prediction analyses (Fig. 3). The average variance explained by multi-PGS was 11.1% for SCA and 4.4% for SCA.g. The likely reason is that the most powerful and predictive PGS in all the multi-PGS analyses are from GWA studies of the highly general traits of educational attainment (EA4) [22], cognitive performance [20], and intelligence (IQ3) [49]. These PGS are highly g-loaded, so their predictive power would be expected to diminish for g-corrected SCA. Our multi-PGS analyses also included PGS from GWA studies of more specific measures of SCA, which added some significant independent prediction of SCA corrected and uncorrected for g in our elastic net analyses. For instance, the PGS for executive function [53] was the second most predictive PGS for mathematics corrected and uncorrected for g. Therefore, although the predictive power is modest for most of the SCA-related PGS included in our analyses, they add to the overall variance explained in our multi-PGS analyses. Importantly, these SCA-related PGS were derived from GWA studies with substantially smaller sample sizes than the most predictive PGS.

We hope these findings encourage more GWA studies of SCA and, in particular, SCA.g. Our twin and SNP heritability estimates indicate that SCA, both corrected and uncorrected for g, are good targets for genomic prediction. In addition, the multi-PGS approache indicate that existing PGS of SCA add to the prediction of both SCA and SCA.g – even in models that include powerful predictors from general traits.

The average results for SCA mask some interesting findings for the individual SCA. For example, reading is significantly less g-loaded compared to mathematical and language ability. Despite the limitations of the extant PGS used in our multi-PGS analyses, they predicted a substantial proportion of the variance for SCA.g for reading (6.9%), but less so for mathematics (3.6%) and language ability (2.5%). The twin heritability results support these PGS findings. Mathematics and language ability have a greater drop in twin heritability (from 50.6% to 34.9%, and from 42.5% to 23.6%, respectively) as compared to a drop from 67.0% to 62.1% for reading. Although these results suggest that reading is less g-dependent than mathematics and language, caution is warranted because a common pathways twin model-fitting analysis of these data reported that the genetic correlation between latent factors representing reading and g is 0.88, similar to the genetic correlations of 0.86 for mathematics and 0.91 for language [27].

In summary, these results provide further evidence for the substantial heritability of SCA.g and provide the first multi-PGS prediction of cognitive abilities independent of g. The results hopefully mark the beginning towards creating PGS for SCA.g that can be used to create genomic profiles of strengths and weaknesses of abilities without the influence of g. This would allow for a more targeted educational system. For example, if genomic strengths of a child were identified for a cognitive skill, interventions can be developed to nurture the skill from an early age because polygenic scores do not change across development. Similarly, if genomic weaknesses were identified, interventions can be implemented before problems emerge in school. However, in order to create SCA.g PGS with sufficient power to be practically useful, GWA studies of SCA.g with samples in the hundreds of thousands are required.

It is daunting to think about creating GWA studies with these sample sizes that include test data for multiple SCA as well as g, which would be needed to investigate SCA.g. Cognitive assessments are time consuming and costly to administer, especially with the sample sizes required to create powerful predictors of SCA.g. However, a cost-effective solution is to create brief but psychometrically valid measures of SCA that can be administered to the millions of people participating in ongoing biobanks for whom genomic data are available. For example, a gamified 15-minute test has been created to assess verbal ability, non-verbal ability, and g [54]. This approach could be extended to assess other SCA and SCA.g. In the meantime, it is possible to use summary statistics from separate GWA studies of SCA and of g using GWAS-by-subtraction to isolate genetic effects on each SCA independent of g [55]. We are currently conducting GWAS-by-subtraction analyses using extant GWA summary statistics from large GWA samples [17, 19] to create PGS for SCA and SCA.g.

Another option is to create PGS from GWA studies of self-reported measures of SCA and g. Because cognitive tests are usually time consuming and costly, self-report measures could be a viable alternative [56]. For example, a large GWA analysis of self-reported math ability (n = 564,698) and the highest math class taken (n = 430,445) was conducted with participants from 23andMe [20]. The derived PGS predicted an average of 6.2% of the variance of math GPA in an independent sample. Unfortunately, due to the proprietary restrictions of 23andMe, we could only include the top 10,000 SNPs in our PGS derived from the GWA analysis of self-reported highest math class taken [20]. This could be why the PGS for the highest math class taken was not a strong independent predictor in our multi-PGS models for mathematics uncorrected and corrected for g.

### Limitations

The usual limitations of the twin method apply here [3], as well as the typical limitations of PGS and GCTA analyses such as issues related to conducting genomic analyses limited to additive effects of the common SNPs genotyped on SNP arrays.

Although our sample is representative of the UK population for family socio-economic status and ethnicity, the generalizability of our results may be limited to similar populations [26]. In addition, because the TEDS sample is predominantly white, only participants of white ethnic origin were genotyped and therefore included in our analyses. This means that our findings are largely only generalisable to other white populations. GWA analyses using participants from other ancestral populations are needed.

## Conclusion

The average twin heritability estimate of 40% and SNP heritability estimate of 26% for g-corrected mathematical, reading and language ability at age 12 provides further evidence that the heritability of SCA is not merely a reflection of the genetic influence of g. Although we found a substantial decrease in variance explained by multi-PGS approaches for g-corrected SCA compared to uncorrected SCA, this decrease is likely due to the fact that the most powerful predictors in the multi-PGS approach were consistently from GWA studies of the extremely general traits of intelligence and educational attainment. Nonetheless, we were able to predict up to 6.9% of g-corrected cognitive abilities from DNA alone. We hope these results encourage researchers to conduct more GWA studies of SCA, especially SCA.g, that can be used to predict PGS profiles of SCA strengths and weaknesses independent of g.

## Supplementary information

Supplementary Material Table S1, S5, S6, S7, S8, and Figure S1

Supplementary Material Table S2

Supplementary Material Table S3

Supplementary Material Table S4

Supplementary Material Table S9

Supplementary Material Table S10

Supplementary Material Table S11

Supplementary Material Table S12

## Data Availability

Data for this study was sourced from the Twins Early Development Study (TEDS). For detailed information on accessing TEDS data, please visit: https://www.teds.ac.uk/researchers/teds-data-access-policy
