# Supplementary material for: Multi-polygenic score prediction of mathematics, reading, and language abilities independent of general cognitive ability
Source: Mol Psychiatry. 2024 Jul 31;30(2):414–22. doi: 10.1038/s41380-024-02671-w (PMC11746139; doi:10.1038/s41380-024-02671-w)
Supplement: Supplementary file 1 — Supplementary Material Table S1, S5, S6, S7, S8, and Figure S1 [file 41380_2024_2671_MOESM1_ESM.docx]

**Supplementary Materials**

Table S1, S5, S6, S7, S8 and Figure S1

**Supplementary Table S1:** Sample sizes of the twin and genomic analyses

p.2

**Supplementary Table S5:** Means and standard deviations of all the measures by sex and zygosity

p. 3

**Supplementary Table S6:** Analysis of variance (ANOVA) by sex and zygosity for all the measures

p. 4

**Supplementary Table S7:** Twin model fitting results for univariate analysis of g-corrected and uncorrected SCA

p.5

**Supplementary Table S8:** Intraclass correlations and estimated genetic and environmental effects using Falconer’s formula for all the measures

p.5

**Supplementary Figure S1:** Phenotypic correlational matrices between all measures

p. 6 - 7

**Supplementary Table S1.**

Sample sizes of the twin and genomic analyses

1. **Table S1a.**

Sample sizes of the twin analyses

|  | **N twin pairs** | **MZ twin pairs** | **DZ twins pairs** |
| --- | --- | --- | --- |
| Reading | 3050 | 1149 | 1901 |
| Reading.g | 2760 | 1045 | 1715 |
| Mathematics | 3566 | 1323 | 2243 |
| Mathematics.g | 2926 | 1101 | 1825 |
| Language | 3057 | 1144 | 1913 |
| Language.g | 2996 | 1125 | 1871 |
| g | 3170 | 1199 | 1971 |

*Note.* Number (N) of twin pairs per specific cognitive ability (SCA). (Reading, Mathematics and Language ability = uncorrected SCA; Reading.g, Mathematics.g and Language.g = g-corrected SCA. g = general cognitive ability).

1. **Table S1b.**

Sample sizes of the genomic analyses

|  | N individuals (all genotyped) | N individuals (one genotyped individual per twin pair) |
| --- | --- | --- |
| Reading | 3781 | 2399 |
| Reading.g | 3447 | 2185 |
| Mathematics | 4494 | 2845 |
| Mathematics.g | 3666 | 2328 |
| Language | 3799 | 2428 |
| Language.g | 3732 | 2383 |
| g | 3957 | 2519 |

*Note.* Number (N) of individuals per specific cognitive ability (SCA). (Reading, Mathematics and Language ability = uncorrected SCA; Reading.g, Mathematics.g and Language.g = g-corrected SCA. g = general cognitive ability).

**Supplementary Table S5.**

Means and standard deviations of all the measures by sex and zygosity

|  | | **Female** | | | **Male** | | | **MZ** | | | **DZ** | |
| --- | --- | --- | --- | --- | --- | --- | --- | --- | --- | --- | --- | --- |
| **SCA** | **M** | | **SD** | **M** | | **SD** | **M** | | **SD** | **M** | | **SD** |
| Reading | 0.02  (3429) | | 0.98 | -0.03  (2658) | | 1.02 | -0.06  (2278) | | 1.00 | 0.04  (3809) | | 1.00 |
| Reading.g | 0.08  (3138) | | 0.98 | -0.10  (2378) | | 1.02 | -0.02  (2076) | | 1.00 | 0.01  (3440) | | 1.00 |
| Mathematics | -0.07  (3943) | | 1.00 | 0.09  (3151) | | 0.99 | -0.04  (2628) | | 1.01 | 0.02  (4466) | | 1.00 |
| Mathematics.g | -0.02  (3334) | | 1.00 | 0.03  (2503) | | 1.01 | 0.00  (2193) | | 1.00 | 0.00  (3644) | | 1.00 |
| Language | 0.03  (3451) | | 1.00 | -0.03  (2641) | | 1.00 | -0.06  (2295) | | 1.00 | 0.03  (3797) | | 1.00 |
| Language.g | 0.1  (3393) | | 0.98 | -0.13  (2578) | | 1.01 | -0.01  (2249) | | 1.00 | 0.01  (3722) | | 1.00 |
| g | -0.08  (3591) | | 0.99 | 0.10  (2749) | | 1.00 | -0.08  (2395) | | 0.99 | 0.05  (3945) | | 1.00 |

*Note.* SCA = specific cognitive abilities. (Reading, Mathematics and Language ability = uncorrected SCA; Reading.g, Mathematics.g and Language.g = g-corrected SCA. g = general cognitive ability). M = mean; SD = standard deviation; MZ = monozygotic twins; DZ = dizygotic twins. The means and standard deviations for the twin analyses were conducted using data from all available twins. The sample sizes are shown in brackets under the mean estimates and they display the number of individuals in the analyses.

**Supplementary Table S6.**

Analysis of variance (ANOVA) by sex and zygosity for all the measures

|  |  |  |  |  |  |
| --- | --- | --- | --- | --- | --- |
| **SCA** | **Sex** | **Zygosity** | **Sex*Zygosity** | **R^2^** | **N** |
| Reading | 0.67 | 0.00* | 0.26 | 0.00 | 3050 |
| Reading.g | 0.00* | 0.89 | 0.21 | 0.00 | 2760 |
| Mathematics | 0.00* | 0.21 | 0.72 | 0.01 | 3566 |
| Mathematics.g | 0.05 | 0.24 | 0.84 | 0.00 | 2926 |
| Language | 0.02 | 0.07 | 0.35 | 0.00 | 3057 |
| Language.g | 0.00* | 0.41 | 0.36 | 0.01 | 2996 |
| g | 0.00* | 0.00* | 0.47 | 0.01 | 3170 |

*Note.* SCA = specific cognitive abilities. (Reading, Mathematics and Language ability = uncorrected SCA; Reading.g, Mathematics.g and Language.g = g-corrected SCA; g = general cognitive ability). Sex = p value; Zygosity = p value; Sex*Zygosity = p value; R^2^ = adjusted R^2^. The ANOVA were conducted using one member from each twin pair. *Significant at α = 0.05 after a Bonferroni correction for multiple testing (p < 0.00714).

**Supplementary Table S7.**

Twin model fitting results for univariate analysis of g-corrected and uncorrected SCA

| SCA | A | C | E |
| --- | --- | --- | --- |
| Reading | 67.00%  (59% - 75%) | 13.60%  (6% - 21%) | 19.41%  (18% - 21%) |
| Reading.g | 62.14%  (51% - 69%) | 3.41%  (0% - 12%) | 34.46%  (31% - 38%) |
| Mathematics | 50.60%  (42% - 59%) | 19.49%  (12% - 27%) | 29.91%  (27% - 33%) |
| Mathematics.g | 34.93%  (21% - 47%) | 8.09%  (0% - 18%) | 56.98%  (52% - 62%) |
| Language | 42.50%  (33% - 52%) | 24.31%  (16% - 32%) | 33.18%  (30% - 36%) |
| Language.g | 23.58%  (12% - 38%) | 12.77%  (2% - 23%) | 63.65%  (58% - 69%) |

*Note.* SCA = specific cognitive abilities. (Reading, Mathematics and Language ability = uncorrected SCA; Reading.g, Mathematics.g and Language.g = g-corrected SCA). 95% confidence intervals shown in brackets.

**Supplementary Table S8**

Intraclass correlations and estimated genetic and environmental effects using Falconer’s formula for all the measures

| SCA | rMZ | rDZ | A | C | E |
| --- | --- | --- | --- | --- | --- |
| Reading | 0.80 | 0.46 | 0.67 | 0.13 | 0.20 |
| Reading.g | 0.65 | 0.33 | 0.64 | 0.01 | 0.35 |
| Mathematics | 0.70 | 0.44 | 0.52 | 0.18 | 0.30 |
| Mathematics.g | 0.43 | 0.26 | 0.34 | 0.09 | 0.57 |
| Language | 0.66 | 0.45 | 0.43 | 0.23 | 0.34 |
| Language.g | 0.36 | 0.24 | 0.24 | 0.12 | 0.64 |
| g | 0.68 | 0.45 | 0.46 | 0.22 | 0.32 |

*Note.* SCA = specific cognitive abilities. (Reading, Mathematics and Language ability = uncorrected SCA; Reading.g, Mathematics.g and Language.g = g-corrected SCA). rMZ = correlation between monozygotic twins; rDZ = correlation between dizygotic twins, A = additive genetic component, C = shared environmental component, E = non-shared environmental component. The ACE estimates were calculated using Falconer’s estimate, which assumes an additive model in which genetic relatedness is 100% for MZ twins and 50% for DZ twins. Thus, A is calculated as 2(rMZ-rDZ), C is estimated as residual MZ resemblance not explained by A (rMZ – A) and E is the remaining variance (1 – rMZ).

**Supplementary Figure S1.**

Phenotypic correlational matrices between all measures

1. **Figure S1a.** Phenotypic correlations between all the measures in the twin analyses

*Note.* Reading, Mathematics and Language ability = uncorrected SCA; Reading.g, Mathematics.g and Language.g = g-corrected SCA; g = general cognitive ability.

1. **Figure S1b.** Phenotypic correlations between all the measures in the genomic analyses

*Note.* Reading, Mathematics and Language ability = uncorrected SCA; Reading.g, Mathematics.g and Language.g = g-corrected SCA; g = general cognitive ability.
